# Applying a clinical staging model in patients affected by schizophrenia spectrum disorder

**DOI:** 10.3389/fpsyt.2024.1387913

**Published:** 2024-07-16

**Authors:** Renato de Filippis, Elvira Anna Carbone, Marianna Rania, Matteo Aloi, Cristina Segura-Garcia, Pasquale De Fazio

**Affiliations:** ^1^ Psychiatry Unit, Department of Health Sciences, University Magna Graecia of Catanzaro, Catanzaro, Italy; ^2^ Outpatient Unit for Clinical Research and Treatment of Eating Disorders, University Hospital Renato Dulbecco, Catanzaro, Italy; ^3^ Department of Clinical and Experimental Medicine, University of Messina, Messina, Italy; ^4^ Psychiatry Unit, Department of Medical and Surgical Sciences, University Magna Graecia of Catanzaro, Catanzaro, Italy

**Keywords:** early intervention in psychosis, phases, predictors, psychosis, schizophrenia, staging

## Abstract

**Background:**

Clinical staging, already widespread in medicine, represents a new frontier in psychiatry. Our goal was to convert the existing theoretical staging model for schizophrenia into a feasible tool to have a timely assessment of patients’ health status applicable in any psychiatric facility.

**Methods:**

We assessed the empirical soundness of a staging model for schizophrenia spectrum disorders (SSDs), primarily centered on their current status. This model delineated six sequential stages (1, 2A, 2B, 3A, 3B, and 4) based on factors like symptom recurrence, persistence, and progression, including functional decline. Our analysis involved data from 137 individuals affected by SSDs. We examined 22 baseline variables, 23 construct-related variables, and 31 potentially modifiable clinical variables.

**Results:**

The latter stages demonstrated significantly poorer outcomes compared to the early stages across various measures, indicating medium to large effect sizes and a dose–response pattern. This pattern confirmed the validity of the model. Notably, stages 2 and 3A exhibited pronounced differences in comparison to other stages, although variables from each validation category also distinguished between consecutive stages, particularly 3A and beyond.

**Conclusion:**

Baseline predictors, such as familial predisposition to schizophrenia, neurodevelopmental impairment, childhood adversities, treatment delay, negative symptoms, neurological impairment, and inadequate early response to treatment, independently largely explained the staging variance. The clinical staging model, grounded in the extended course of psychosis, exhibited sound validity and feasibility, even without the use of biological or neuroimaging markers, which could greatly improve the sensitivity of the model. These findings provide insights into stage indicators and predictors of clinical stages from the onset of psychosis.

## 1 Introduction

In the ever-evolving landscape of medical research and practice, the application of clinical staging models has become a pivotal paradigm, notably flourishing in fields like oncology and cardiology (1). This approach, which categorizes illnesses into distinct stages according to their progression, severity, and underlying biological alterations, has demonstrated its effectiveness in directing treatment approaches and enhancing patient outcomes (2).

Clinical staging diverges from traditional diagnostic methods as it not only delineates the extent of illness severity but also situates a patient within the course of the disorder (3). This introduces the concept of early interventions to enhance recovery in the disease’s early phases, to prevent progression to later stages, and to move towards treatment personalization (3–5). While clinical staging as a model for categorizing the development of disorders was previously overlooked in psychiatry, several theories have surfaced in recent decades for major disorders (1, 2, 6).

Indeed, traditionally, psychiatry has preferred a categorical and more static classification approach to a dimensional and more dynamic one, to define mental disorders, providing clinicians with a consensus-based framework for diagnosis and treatment, derived from the main classification tools (7, 8). Nevertheless, the inherent dynamism and clinical heterogeneity of schizophrenia (SCZ) present notable challenges to these conventional traditional models (9, 10). Hence, recognizing the shortage of static categorizations to capture the evolving nature of SCZ has prompted a shift in focus towards adopting a clinical staging approach (11, 12).

The inaugural staging model arose in 1982, and subsequent decades have witnessed the development of various clinical staging concepts for several psychiatric disorders (1, 2, 13). Despite the absence of a clinical consensus designating a gold standard among these models, integrating them reveals an overarching, unified staging concept comprising four distinct stages for SCZ (14–17).

Based on the information provided, it can be inferred that the clinical staging concept commonly employed in other medical disciplines could potentially be relevant to mental and behavioral disorders (1, 2, 4). In particular, several theoretical staging models have been formulated for conditions such as bipolar disorder, depression, and SCZ over the past decade. While there are promising data for the staging of bipolar disorder (18–21), only one of these models, specifically designed for depression, has demonstrated empirical validity (22, 23). Consequently, despite ongoing efforts, it is crucial to either validate the existing models or develop new empirical staging models that have proven validity for regular clinical application.

To address this gap, our study aimed to stage psychotic disorders using a real-world clinical sample of individuals suffering from schizophrenia spectrum disorders (SSDs), with a cross-sectional observation. The primary objective was to investigate whether empirical evidence could support a staging model based on the clinical severity of psychotic disorders.

Specifically, we sought to apply a staging model for well-established psychosis, incorporating the principles of clinical staging and drawing from existing literature on the staging of psychotic disorders to a cohort of participants affected by SSDs.

## 2 Materials and methods

### 2.1 Participants and procedures

We included adults suffering from SSDs and consecutively admitted from July 2020 to May 2023 to the outpatient Psychiatry Unit of the University Hospital Magna Græcia of Catanzaro (Italy), through a cross-sectional evaluation. Eligibility criteria for participants included the following: (1) age between 18 and 75 years with the ability to provide informed consent; (2) a diagnosis of SCZ or SSD confirmed by a senior psychiatrist using the Diagnostic and Statistical Manual of Mental Disorders (DSM-5) diagnostic criteria (8); and (3) regular visits to the Unit for a minimum of 12 consecutive months. Clinical diagnosis was conducted using the Structured Clinical Interview for DSM-5 (SCID-5-CV) (24) by an experienced psychiatrist trained in neuropsychiatric examinations, adhering to DSM-5 criteria (8). Exclusion criteria encompassed the following: (1) a diagnosis of dementia, intellectual disability, or other severe medical conditions associated with secondary psychiatric symptoms that could potentially bias the assessment; (2) a diagnosis of substance use disorder within the last 12 months; (3) inability to complete the assessment due to conditions like speech impairments or a lack of proficiency in the Italian language; and (4) an invalid informed consent for the study procedures.

Before data collection, the study protocol underwent review and approval by the local Ethical Committee of University Hospital Mater Domini of Catanzaro (Italy), “Regione Calabria, sezione Area Centro” (n. 191/2020). The study procedures and protocol adhered to ethical principles outlined in the latest version of the Helsinki Declaration (25), and all patients provided written informed consent in accordance with the ethical committee’s guidelines before data collection commenced.

### 2.2 Measures

#### 2.2.1 Clinical and sociodemographic characteristics

An *ad hoc* designed schedule was employed to collect the demographic and clinical features of the sample participants. The collected data encompassed factors such as psychiatric personal and family history, age at the onset of illness, duration of untreated psychosis (DUP), prior psychiatric hospitalizations (number, type, and duration), suicide attempts, previous psychotherapy, antipsychotic treatments (number, type, and formulation), and number of psychotic episodes.

#### 2.2.2 Assessment

All participants were evaluated by means of the following tests during the enrollment visit: (1) the Childhood Trauma Questionnaire Short-Form (CTQ-SF) (26); (2) the Positive and Negative Symptom Scale (PANSS) (27); (3) the Aberrant Salience Inventory (ASI) (28); (4) the Global Assessment of Functioning (GAF) scale (29); (5) the Personal and Social Performance (PSP) scale (30); and (6) the Quality of Life (QoL) scale (31).

In summary, we evaluated construct validators of clinical staging over the course of the illness from the first episode of psychosis (FEP) to the follow-up assessment visit, considering factors like DSM-5 diagnosis, neurological abnormalities and general medical comorbidities, illness-extension variables, and mental health service utilization. Outcome validators were also appraised during the evaluation, encompassing childhood trauma and adversities (i.e., CTQ), psychopathology (i.e., PANSS and ASI), functioning (i.e., GAF and PSP), and self-rated quality of life (i.e., QoL scale). To assess the clinical validity of baseline variables for the final staging model, we examined baseline variables recorded at the FEP, including family history, distal antecedents, intermediate (premorbid) antecedents, proximal antecedents, illness-onset features, index-episode characteristics, and variables assessed during the visit.

#### 2.2.3 Clinical staging

The most used clinical staging model for psychotic disorders extends from stage 0 to stage 4, commencing with an at-risk yet asymptomatic state (stage 0) and progressing in severity to nonspecific symptoms or an attenuated syndrome (stage 1), a full-threshold disorder (stage 2), recurrence and persistence of illness (stage 3), and severe, unremitting illness (stage 4) (32), with subsequent slight changes to the original model having been suggested (33).

In the definition of stages, we employed the general staging framework established by McGorry (32, 34), together with the recent clinical characterization of patients affected by primary psychosis proposed by Maj et al. (35) and the application model envisaged by the recent validation study by Peralta and colleagues adapted to a cross-sectional evaluation (36). Thus, stages have been determined based on the history of disease, symptoms severity, and declining functioning (see **Supplementary Table 1**). Hence, we identified four main stages: stage 1 (mild/moderate psychotic symptoms), stage 2 (episodic course with full remission), stage 3 (episodic course with partial remission), and stage 4 (chronic/continuous course). These primary stages were further divided into the following sub-stages: stage 2A (single episode with full remission), stage 2B (multiple episodes with full remission), stage 3A (episodic course with partial and stable remission), and stage 3B (episodic course with partial remission and progressive course).

It is important to observe some notable distinctions from other staging models. Firstly, our model does not consider pre-psychotic phases of illness or at-risk mental state (ARMS) (37) and concentrates on established psychosis (stages 1–4), thus excluding stage 0 or the at-risk but asymptomatic phase, of which we acknowledge the utility, and which needs further studies to be explored in detail. Secondly, in our model, episodes with full remission, a desirable treatment outcome, takes precedence over recurrence with partial remission and is thus classified within stage 2A rather than stage 2B or 3, as previously applied in similar research (36). This acknowledges the significant proportion of non-SCZ psychotic episodes that may have a self-remitting character (38, 39). Thirdly, stages 2B and 3B were included as recognized course patterns in both clinical practice and long-term follow-up studies (36, 40), also existing staging models in the McGorry classification (32).

**Figure 1:**
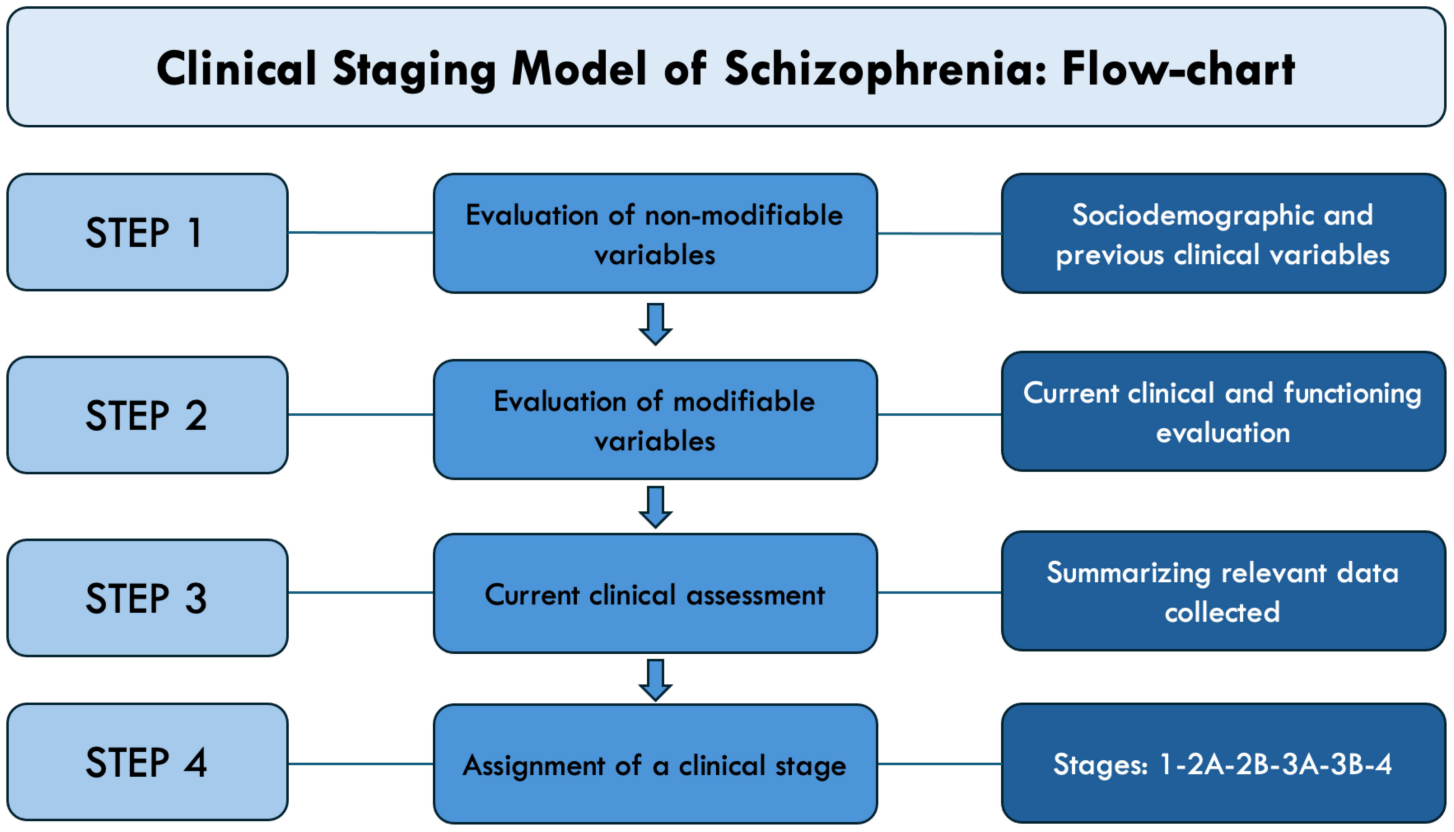
Clinical staging assignment flowchart.

Therefore, we determined staging levels through a follow-back methodology, considering all accessible information such as demographic, health, and social records, as well as conducting interviews with the subject and relevant individuals during the follow-up assessment (**Figure 1**; **Supplementary Material 3**).

### 2.3 Statistical analysis

Data were analyzed using the Social Sciences Statistical Package, Version 26.0 (SPSS Inc., Chicago, IL, USA), and data were presented as means, standard deviations, frequencies, and percentages, as appropriate. In order to select appropriate methods of statistical analysis, the normal distribution was checked using the Shapiro–Wilk test. Mean differences between stages according to psychopathological variables were compared using an analysis of variance (ANOVA) test. The statistical significance level was set at *p* < 0.05 (two-sided).

## 3 Results

### 3.1 Sample characteristics

Out of the initial cohort (*N* = 143), nine (6.3%) individuals were deemed ineligible for inclusion in the study due to various reasons. Of these, three (2.1%) patients displayed disinterest in participating, two patients (1.4%) were not eligible because of active substance use disorder, two (1.4%) met the exclusion criteria of intellectual disability, one participant (0.7%) withdrew from the study before the conclusion of the assessment, and one (0.7%) was unable to read and write. As a result, the final sample comprised 137 participants, with 92 individuals diagnosed with SCZ, 19 with schizoaffective disorder, 16 with unspecified SSD and other psychotic disorders, and 10 with delusional disorder. All participants were recruited sequentially over the course of a 3-year practical clinical investigation. Additionally, the remarkably low rate of patient attrition (i.e., 6.2%) and an impressive complete response rate exceeding 93.8% reflect the robust acceptance of the evaluation methodologies among the participants and their feasibility in everyday clinical practice.

**Table 1** presents the sociodemographic and clinical characteristics of the sample. The final sample included 87 patients (63.5%) who identified themselves as male and 50 (36.5%) who identified as female. The mean age of the participants was 47.5 ± 13.9 years. Most of the sample was single (*n* = 108, 78.8%) and unemployed (*n* = 48, 35.0%) with a mean of previous psychiatric hospitalizations of 1.4 ± 3.4 months, 81 participants (59.1%) were smokers, and 33 (24.1%) were previously affected by drug addiction. Many participants (*n* = 48, 35.0%) reported no familial psychiatric history, while 78 (56.9%) had family cases of affective disorders, and 10 (7.3%) had previous SCZ diagnosis within parents or relatives. The mean disease duration was 21.1 ± 11.8 years, and the mean age of onset of the disorder was 26.1 ± 10.1 years.

**Table 1:** Demographic and clinical characteristics of the sample.

		Frequency	%
**Sex**	Female	50	36.5
	Male	87	63.5
**Age^§^**		47.5	13.9
**Ethnicity**	White	132	96.4
	Black	2	1.5
	Mixed	3	2.2
**Marital status**	Single	108	78.8
	Married	20	14.6
	Divorced	9	6.6
**Work**	Unemployed	48	35.0
	Employed	13	9.5
	Housewife	8	5.8
	Student	9	6.6
	Retired	14	10.2
	Disable	43	31.4
	Self-worker	2	1.5
**Education**	Illiterate	1	0.7
	Elementary school	14	10.2
	Middle school	62	45.2
	High school	46	33.6
	University degree	14	10.2
**Alcohol abuse (yes)**		47	34.3
**Smoke (yes)**		81	59.1
**Drug abuse (yes)**		33	24.1
**Diagnosis**	Schizophrenia	92	67.2
	Schizoaffective disorder	19	13.9
	Unspecified schizophrenia spectrum and other psychotic disorder	16	11.7
	Delusional disorder	10	7.3
**Family history of psychiatric diseases**	None	48	35.0
	Affective disorder	78	56.9
	Schizophrenia	10	7.3
	Personality disorder	1	0.7
**Family history of psychosis**	Yes	22	16.1
**Previous hospitalizations**	Yes	46	33.6
**Hospitalization numbers^§^**		0.8	1.8
**Hospitalization time (months) ^§^**		1.4	3.4
**Compulsory hospitalization**		1.1	1.6
**Suicide attempts**	Yes	13	9.5
**Psychotherapy**	Yes	23	16.8
**Pharmacological treatment**	Yes	129	94.2
**Long-acting injectable antipsychotics**	Yes	87	63.5
**Age at onset^§^**		26.1	10.1
**Disease duration (years) ^§^**		21.1	11.8
**Suicide attempts^§^**		0.1	0.5
**Psychotic episodes^§^**		6.8	4.6

^§^Data reported as mean and standard deviation.

Assessment results are reported in **Table 2**. The mean scores of the scales were as follows: PANSS total score, 118.4 ± 40.9; GAF score, 49.2 ± 13.5; CTQ score, 55.9 ± 9.8; ASI score, 18.7 ± 6.2; QoL total, 42.3 ± 19.7; and PSP, 47.7 ± 13.8.

**Table 2:** Results of psychopathological and functional assessments.

	Mean	Standard deviation	Minimum	Maximum
**PANSS positive**	17.6	8.6	7	41
**PANSS negative**	21.0	9.3	7	42
**PANSS general**	42.5	10.9	22	78
**PANSS total**	118.4	40.9	40	161
**GAF**	49.2	13.5	22	85
**CTQ**	55.9	9.8	28	83
**ASI**	18.7	6.2	2	29
**QoL scale**	42.3	19.7	18	100
**PSP scale**	47.7	13.8	13	77

ASI, Aberrant Salience Inventory; CTQ, Childhood Trauma Questionnaire; GAF, Global Assessment of Functioning; PANSS, Positive and Negative Symptom Scale; PSP, Personal and Social Performance; QoL, Quality of Life.

**Figure 2:**
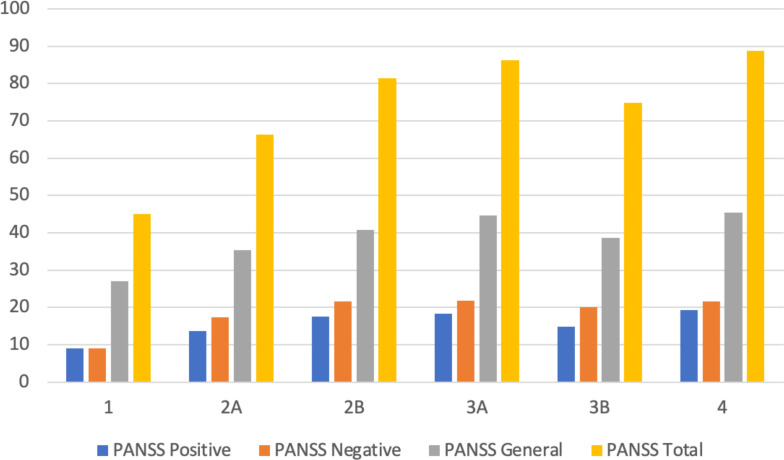
Mean values of PANSS total and subscale scores across clinical stages. PANSS, Positive and Negative Symptom Scale.

**Figure 3:**
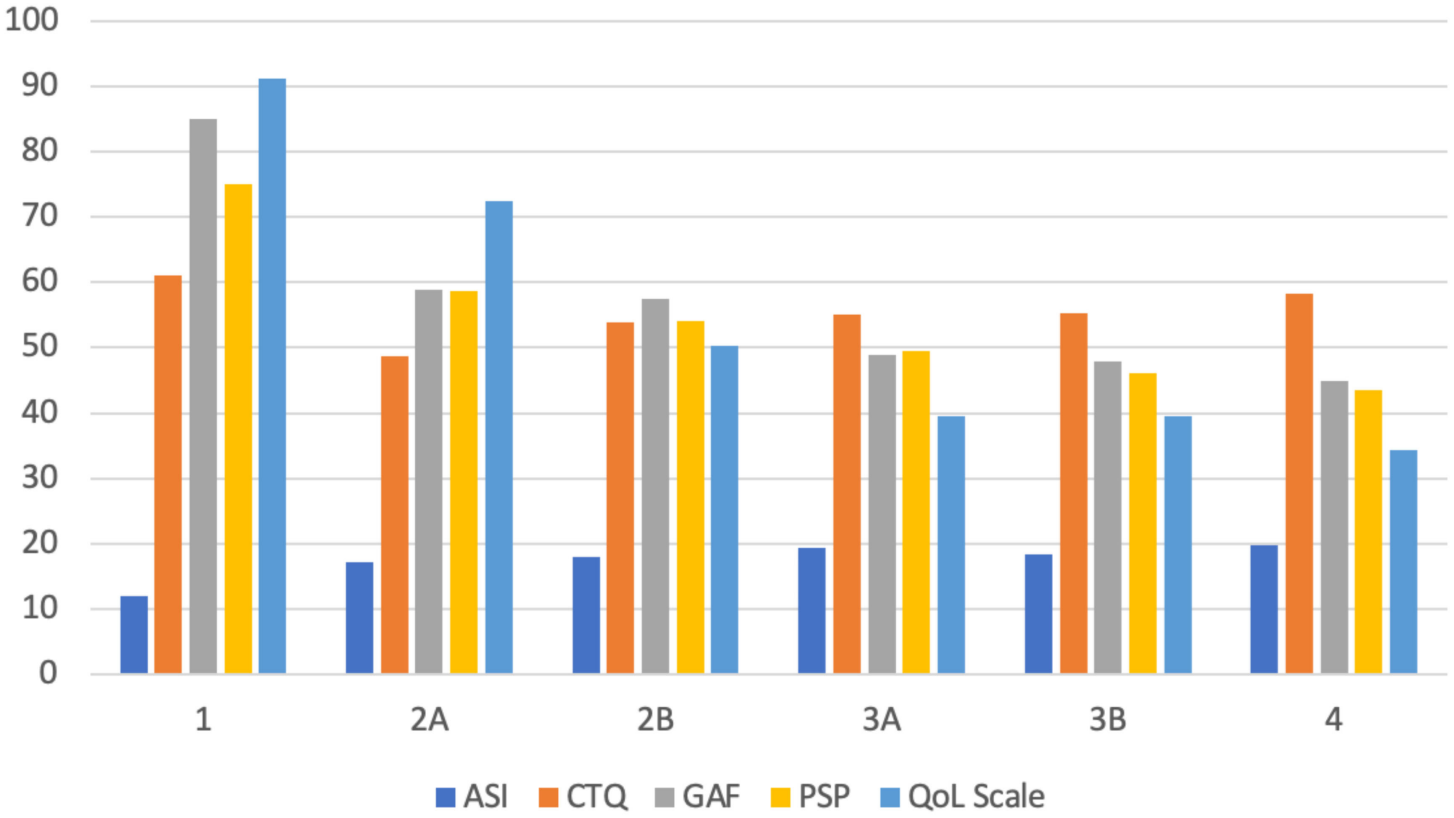
Mean values of ASI, CTQ, GAF, PSP, and QoL scores across clinical stages. ASI, Aberrant Salience Inventory; CTQ, Childhood Trauma Questionnaire; GAF, Global Assessment of Functioning; PSP, Personal and Social Performance; QoL, Quality of Life.

**Table 3** presents the stratified distribution of patients in the different stages according to the clinical characteristics collected. **Figure 2** displays the relationship between the PANSS subscale scores and the total score in relation to clinical stages. **Figure 3** displays the results of ASI, CTQ, PSP, and GAF related to clinical stages. The mean scores and the comparisons between the stages are reported in **Supplementary Table 2**.

**Table 3:** Frequencies of individuals across clinical stages.

Stage	Frequencies	Percentage
**1**	4	2.9
**2A**	9	6.6
**2B**	18	13.1
**3A**	29	21.2
**3B**	27	19.7
**4**	50	36.5

## 4 Discussion

Our study aimed to apply a clinical staging for SCZ based on the current main theoretical model to pave the path to verify its empirical support in clinical practice. Our methodology aligns with practices observed in other medical domains, where staging is grounded in a robust comprehension of the natural progression of illness, refined through the utilization of valid staging subtypes, and individual risk factors to formulate subject-specific predictions regarding illness risk, progression, and overall prognosis. To the best of our knowledge, this assessment stands as the sole evidence-based study explicitly evaluating a theoretical staging model for psychotic disorders, primarily derived from their current state, functioning, and outcomes.

The two main diagnostic systems in psychiatry, viz, the International Classification of Diseases version 11 (ICD-11) (41) and the Diagnostic and Statistical Manual of Mental Disorders version 5 text revised (DSM-5-TR) (42), although currently indispensable for both educational and research purposes and clinical diagnosis and economic/insurance reasons, still exhibit considerable limitations to capture the dynamic complexity of severe psychiatric syndromes and their psychopathological dimensions. Additionally, as new classification methods rooted in neuroscience and genomics evolve, there is a notable gap in the integration of crucial concepts like clinical staging, neuroinflammation, and thorough consideration of endophenotypes within both the DSM and ICD (43).

It is essential to recognize that our model differs from existing staging models of psychosis, albeit remaining within the framework of the model designed by McGorry (3, 32) and with clinical specifications introduced by Maj and colleagues (35). In particular, previous models do not provide assessment indications to be used sequentially to quantify. Conversely, our model predominantly focuses on the extended outcomes objectively assessable by the clinician through medical history collection, clinical interview, and administration of validated tests, and it further distinguishes between stable and progressing courses within the realm of non-remitting psychoses.

Particularly interesting is the role that age at onset, age at the time of assessment, and duration of illness play in the staging of SCZ. Indeed, time could be intuitively interpreted as the main determining factor in the staging of SCZ and in the overall increasing severity of psychosis since significant evidence supports the neurodegenerative hypothesis, particularly concerning the interaction between SCZ and aging (44). On this regard, there are data that correlate SCZ, like many other neuropsychiatric disorders, with accelerated brain aging (45, 46). Therefore, it may be speculated that clinical staging studies with prospective designs correlate the advancing age of patients and the duration of the disease with increasing staging (36). This perspective, however, appears limited as it does not account for the potential for improvement and recovery that does occur. Therefore, the model applied in this study also considered a cross-sectional design, giving less emphasis to the current age and duration of illness. Instead, it focused on the current and specific situation, which can be modified by therapy and rehabilitation (47).

When comparing the characteristics between different stages, it is interesting to note a clear difference in general psychotic symptomatology, as assessed through the PANSS general, which is not equally captured by the positive and negative symptom subscales. This difference becomes even more pronounced during the transition from stage 2A to 2B [i.e., from the “Episodic course with full remission (single episode)” to the “Episodic course with partial remission (multiple episodes)”]. This is unsurprising, as the repetition of several psychotic episodes leads to a notable worsening of the patient’s clinical condition. The chronic-relapsing course of SCZ results in a progressive worsening of symptoms, individual functioning, recovery capacity, and morphological and functional brain activity after each relapse (48, 49). These data support a multi-step, progressive, and prompt staging model as proposed in this work.

Recently, there has been only one attempt to perform clinical staging trials of SCZ, using a different approach with different, albeit comparable, methods and results to our study (36). In this study, Peralta et al. assessed the empirical validity of a staging model for psychotic disorders primarily grounded in their extended course delineating six consecutive stages (2A, 2B, 3A, 3B, 4A, and 4B) based on factors like symptom recurrence, persistence, and progression, including functional decline with data from 243 participants experiencing FEP (36). The authors stated that later stages consistently exhibited significantly poorer outcomes compared to earlier stages across most validators, displaying generally medium to large effect sizes and a dose–response pattern. Actually, our study represents the first real attempt to apply a staging model, albeit modified, to a real population through a prospective analysis and starting from the FEP. Our model has the ambition of being able to be applied to any patient at any time, with a transversal design and with object data from the personal and clinical history and from the psychopathological state of the patient suffering from SSDs.

Still, according to Peralta and colleagues, baseline predictors, such as familial SCZ load, neurodevelopmental impairment, childhood adversities, treatment delay, negative symptoms, neurological impairment, and poor early treatment response, independently explained 49.9% of the variance in staging (36). This result overlaps with our findings, and in some way confirms them by providing the theoretical substrate on which the additional clinical analysis is built. In fact, following the analysis suggested by Maj and colleagues, we also evaluated salient domains to be considered in the clinical characterization of a patient with a diagnosis of primary psychosis, i.e., psychopathological components, onset and course, social functioning, family history, physical and psychiatric comorbidities, childhood trauma and adversities, and protective/risk factors (35).

Therefore, a psychosis staging model primarily based on its long-term course demonstrated robust construct, outcome, and predictive validity. On the other hand, our findings have the potential to enhance our understanding of stage indicators and predictors of clinical stages with a cross-sectional viewpoint and a dynamic application of the model through multiple potentially repeated evaluation. Therefore, the use of rigid and unchangeable classification means, as well as the use of purely categorical diagnostic systems, fails to capture the nuances and dynamism of features inherent in a multifaceted disorder such as SCZ (50).

However, SCZ is a very dynamic and heterogeneous disorder in its clinical presentation. While it is possible to identify a certain cyclicality and progressiveness between the advancement of clinical stages and the clinical and functional worsening, there are also chronic patients with a long history of illness who achieve an excellent level of global recovery (51, 52). Therefore, it appears anachronistic to consider patients more serious solely based on the longer duration of their illness, especially compared to young acute patients with a dramatic decline in post-episode functioning (53). From this perspective, a transversal clinical staging model, possibly applied multiple times over the course of illness, seems better suited to the nature of the disorder than a retrospective or prospective model that does not account for the current nature of the condition.

Finally, the concept of within-stage heterogeneity explains the benefits of defining stages in terms of clinical psychopathology and stage modifiers. Using examples from medicine, we discuss the usefulness of categorizing stage modifiers into factors related to progression (i.e., potential predictors of stage transition) and extension (i.e., factors associated with the current presentation that complicate treatment selection). Finally, we propose revising the current transdiagnostic staging approach to incorporate these key concepts, and we suggest how this revised framework could be applied in both clinical and research settings.

### 4.1 Clinical implications

When considering clinical staging in medicine, it is expected that it can represent a tool that is feasible and practical to use, ubiquitous and potentially able to provide both reliable prognostic assessment and to offer personalized therapeutic strategies within evidence-based guidelines (54–56). While our data do not directly contribute to treatment selection insights, they do furnish vital information for real-time stage allocation and, consequently, potential tailored interventions.

We observed a wide range of baseline demographic and clinical variables associated with staging, providing valuable input for clinicians and researchers in developing prediction models that can identify staging predictors from the onset of psychosis. Therefore, our intent was to apply the theoretical staging model (3, 32), based on high-impact clinical indicators (35), in order to provide a practical tool directly usable by clinicians, which would give a dynamic and flexible indication on the level of severity of the disorder. In this context, significant effects on stage allocation have been identified for factors such as obstetric complications, developmental delay, childhood trauma and adversities, poor psychosocial adjustment, limited cognitive reserve, onset of chronic illness, DUP, negative symptoms, and inadequate early treatment response, as confirmed by previous research (36). This underscores the importance of evaluating these variables in states of heightened risk and in cases of FEP. This emphasizes the importance of evaluating these variables at any time, since the main value of our research is the idea to apply the staging model in different phases of the disease path to prevent the transition to a worse stage or decrease it if it has already occurred. Indeed, while the early intervention at FEP has been a focal point for intervention within the early intervention paradigm aimed at reducing subsequent disability (57, 58), our findings propose that clinical staging may be a more suitable candidate than single-variable evaluation for averting the progression to the most severe stages. Indeed, while many of the baseline predictors of staging identified in this study pose challenges in management due to their pre-existing nature (e.g., familiar history of psychosis), especially as they are established before the onset of illness (e.g., childhood trauma and adversities), they can offer insights into tailoring service provisions for individuals at risk of non-recovery. Furthermore, the gradual evolution of stages over time, primarily driven by transitions from lower to higher severity stages, does not exclude the possibility of downgrade, especially within the first clinical stages, just as it is possible that an individual may be staged in different stages at different times in life, if we consider modifiable risk factors such as treatment adherence, personal relationships, work activity, and income.

On the other hand, identifying risk factors for non-recovery provides an opportunity to address them preemptively, rather than solely after the establishment of non-recovery. Consequently, efforts to develop preventive strategies for later stages should commence as early as possible regardless of the clinical stage (35, 36). Specifically, individuals at risk of incomplete remission after a psychotic episode should be a focal point for attention concerning pharmacological treatment, including exploring the potential benefits of clozapine or early prescription of long-acting injectable (LAI) antipsychotic formulations, as well as engaging in multimodal intensive psychosocial rehabilitation programs (59, 60). In fact, one of the most current topics of debate in the literature is represented by the timing of use, possible therapeutic switch, and the duration of LAI intervention compared to the onset and prognosis of SCZ (61, 62). The routine application of a staging application model for SCZ in clinical practice could allow clinicians to optimize LAI both at the time of a new diagnosis and in the event of relapses or medium- to long-term rehabilitation (63, 64).

In conclusion, one of the main practical applications of a validated clinical staging model could be its use in clinical practice by integrating it with the current main existing pharmacological and rehabilitation guidelines. This would allow for a step-by-step intervention protocol based on current clinical stages, addressing both conditions of improvement and worsening (65–67). Even if such an integration is still under development according to current literature, it is conceivable that new-generation antipsychotic drugs in LAI formulation, together with psychoeducational, psychosocial, and rehabilitative interventions, should be already proposed in the early clinical phases. In the more advanced stages, interventions could then be extended to social support, family involvement, and reintegration into the world of work.

## 5 Limitations and strengths

When interpreting the results of our study, it is relevant to evaluate some limitations and strengths. First, although the overall sample size is adequate to draw global assessments, the single clinical staging levels led to a reduced sample size in certain subgroups, consequently diminishing the statistical power to identify significant differences across stages. Second, the applied clinical and assessment evaluation, despite containing tools widely used and validated in the literature, and although having been used in the appropriate population and for the intended purposes, has not been validated as a single clinical staging evaluation tool before. However, this also represents the peculiar and novel element of this work and is based on evidence-based data and clinical hypotheses that are easily reproducible in other clinical contexts and psychiatric facilities worldwide. Third, the included sample presents a wide sociodemographic and clinical heterogeneity, consistent with real-world practice. Finally, a further limitation of the study concerns the use of a cross-sectional evaluation, which returns a profile of the patient referring to the exact moment in which this is studied. Future studies should be able to carry out this type of clinical staging assessment prospectively, also including patients’ cognitive functioning evaluation.

On the other hand, the choice to apply a cross-sectional study design together with the chance to use easily accessible data from the individual history of patients and assessment tools, which are widely used and available and well-known by mental health professionals, makes this staging protocol easily reproducible and applicable in different contexts. Moreover, this is the first attempt to apply the clinical staging of SCZ in a real-world population, using both retrospective and current information, objective and feasible validated tools, and with a design that allows even a single clinical observation to place patients in a specific clinical stage.

## 6 Conclusion

Usually, clinical staging models place their primary emphasis on delineating distinct stages of the disorder based on severity, progression, and symptom characteristics, aiming to enhance prognostic predictions. From a clinical perspective, the definition of discrete stages establishes a framework for the development of interventions focused on prevention. In this study, we proposed a practical application of clinical staging categorizing 137 outpatients suffering from SSDs starting from the McGorry staging model and then modify it into a clinical adaptation. This classification is intended to assist clinicians in better characterizing patients and selecting treatments tailored to each specific stage. Our results show that a pragmatic use of clinical staging models in clinical practice not only is possible, but also provides a timely assessment of the stage of severity in which the patient is at the observation moment. In the future, it would be desirable to apply these staging models in the earlier stages, where intervention is more effective in curbing the progression of the illness, and with a prospective study design. Moreover, integration with biological, neuroinflammation, and neuroimaging markers is not only desirable but also necessary to improve the sensitivity and precision of a modern staging model of SCZ. Similarly, it will be necessary to dedicate more space to the evaluation of comorbidity with physical diseases and health-related quality of life, as well as social and protective factors, for a more holistic classification potentially integrated into routine clinical practice.

## Data availability statement

The raw data supporting the conclusions of this article will be made available by the authors, without undue reservation.

## Ethics statement

The study was conducted in accordance with the principles of the Declaration of Helsinki and approved by the Ethics Committee of University Hospital Mater Domini of Catanzaro (Italy) ‘Regione Calabria, sezione Area Centro’ (n. 191/2020). The studies were conducted in accordance with the local legislation and institutional requirements. The participants provided their written informed consent to participate in this study.

## Author contributions

RdF: Conceptualization, Investigation, Writing – original draft, Writing – review & editing. EAC: Writing – review & editing. MR: Writing – review & editing. MA: Writing – review & editing. CSG: Formal analysis, Methodology, Writing – review & editing. PDF: Conceptualization, Writing – review & editing.

## Supplementary material

Supplementary Table 1 Clinical staging guidelines.

Supplementary Table 2 Mean differences between stages according to psychopathological variables.

Supplementary Material 3 Methodology to assign the clinical stage.

## References

[1] Cosci F Fava GA. Staging of mental disorders: systematic review. Psychother Psychosom. (2013) 82:20–34. doi: 10.1159/000342243 23147126

[2] Borbála Dombi Z Barabássy Á Sebe B Laszlovszky I Németh G. Clinical staging in schizophrenia spectrum disorders. In: Psychosis - Phenomenology, Psychopathology and Pathophysiology. IntechOpen (2022). doi: 10.5772/intechopen.98276

[3] McGorry PD Nelson B Goldstone S Yung AR. Clinical staging: a heuristic and practical strategy for new research and better health and social outcomes for psychotic and related mood disorders. Can J Psychiatry. (2010) 55:486–97. doi: 10.1177/070674371005500803 20723276

[4] Agius M Goh C Ulhaq S McGorry P. The staging model in schizophrenia, and its clinical implications. Psychiatr Danub. (2010) 22:211–20. 20562749

[5] Arango C Díaz-Caneja CM McGorry PD Rapoport J Sommer IE Vorstman JA. Preventive strategies for mental health. Lancet Psychiatry. (2018) 5:591–604. doi: 10.1016/S2215-0366(18)30057-9 29773478

[6] Verduijn J Milaneschi Y van Hemert AM Schoevers RA Hickie IB Penninx BWJH. Clinical staging of major depressive disorder. J Clin Psychiatry. (2015) 76:1200–8. doi: 10.4088/JCP.14m09272 26455670

[7] World Health organization (WHO). The ICD-10 Classification of Mental and Behavioural Disorders Clinical descriptions and diagnostic guidelines . Available online at: http://www.who.int/classifications/icd/en/bluebook.pdf (Accessed February 19, 2018).

[8] American Psychiatric Association. Diagnostic and Statistical Manual of Mental Disorders: DSM-5. fifth Ed. Washington: American Psychiatric Association (2013). doi: 10.1176/appi.books.9780890425596

[9] Tschacher W Scheier C Hashimoto Y. Dynamical analysis of schizophrenia courses. Biol Psychiatry. (1997) 41:428–37. doi: 10.1016/S0006-3223(96)00039-X 9034537

[10] an der Heiden U. Schizophrenia as a dynamical disease. Pharmacopsychiatry. (2006) 39:36–42. doi: 10.1055/s-2006-931487 16508894

[11] Martínez-Cao C de la Fuente-Tomás L García-Fernández A González-Blanco L Sáiz PA Garcia-Portilla MP. Is it possible to stage schizophrenia? A systematic review. Transl Psychiatry. (2022) 12:197. doi: 10.1038/s41398-022-01889-y 35545617 PMC9095725

[12] de Filippis R Carbone EA Gaetano R Bruni A Pugliese V Segura-Garcia C. Machine learning techniques in a structural and functional MRI diagnostic approach in schizophrenia: a systematic review. Neuropsychiatr Dis Treat. (2019) 15:1605–27. doi: 10.2147/NDT.S202418 PMC6590624 31354276

[13] Brugnoli R Rapinesi C Kotzalidis GD Marcellusi A Mennini FS De Filippis S. Model of Management (Mo.Ma) for the patient with schizophrenia: crisis control, maintenance, relapse prevention, and recovery with long-acting injectable antipsychotics (LAIs). Riv Psichiatr. (2016) 51:47–59. doi: 10.1708/2246.24194 27183509

[14] Hoffman BF. The stages of schizophrenia and their management. Can Fam Physician. (1982) 28:2046–50. PMC2306672 21286547

[15] Fava GA Kellner R. Staging: a neglected dimension in psychiatric classification. Acta Psychiatr Scand. (1993) 87:225–30. doi: 10.1111/j.1600-0447.1993.tb03362.x 8488741

[16] Lieberman JA Perkins D Belger A Chakos M Jarskog F Boteva K. The early stages of schizophrenia: speculations on pathogenesis, pathophysiology, and therapeutic approaches. Biol Psychiatry. (2001) 50:884–97. doi: 10.1016/S0006-3223(01)01303-8 11743943

[17] Singh SP Cooper JE Fisher HL Tarrant CJ Lloyd T Banjo J. Determining the chronology and components of psychosis onset: The Nottingham Onset Schedule (NOS). Schizophr Res. (2005) 80:117–30. doi: 10.1016/j.schres.2005.04.018 15978778

[18] Berk M Post R Ratheesh A Gliddon E Singh A Vieta E. Staging in bipolar disorder: from theoretical framework to clinical utility. World Psychiatry. (2017) 16:236–44. doi: 10.1002/wps.20441 PMC5608827 28941093

[19] Vieta E Reinares M Rosa AR. Staging bipolar disorder. Neurotox Res. (2011) 19:279–85. doi: 10.1007/s12640-010-9197-8 20461491

[20] Duffy A. Toward a comprehensive clinical staging model for bipolar disorder: integrating the evidence. Can J Psychiatry. (2014) 59:659–66. doi: 10.1177/070674371405901208 PMC4304586 25702367

[21] Macellaro M Girone N Cremaschi L Bosi M Cesana BM Ambrogi F. Staging models applied in a sample of patients with bipolar disorder: Results from a retrospective cohort study. J Affect Disord. (2023) 323:452–60. doi: 10.1016/j.jad.2022.11.081 36455717

[22] Fava GA Tossani E. Prodromal stage of major depression. Early Interv Psychiatry. (2007) 1:9–18. doi: 10.1111/j.1751-7893.2007.00005.x 21352104

[23] de la Fuente-Tomas L Sánchez-Autet M García-Álvarez L González-Blanco L Velasco Á Sáiz Martínez PA. Estadificación clínica en los trastornos mentales graves: trastorno bipolar, depresión y esquizofrenia. Rev Psiquiatr Salud Ment. (2019) 12:106–15. doi: 10.1016/j.rpsm.2018.08.002 30314812

[24] First M Williams J Karg R. Structured clinical interview for DSM-5 disorders, clinician version (SCID-5-CV). Am Psychiatr Assoc. (2015).

[25] World Medical Association. World Medical Association Declaration of Helsinki: ethical principles for medical research involving human subjects. JAMA. (2013) 310:2191. doi: 10.1001/jama.2013.281053 24141714

[26] Innamorati M Erbuto D Venturini P Fagioli F Ricci F Lester D. Factorial validity of the Childhood Trauma Questionnaire in Italian psychiatric patients. Psychiatry Res. (2016) 245:297–302. doi: 10.1016/j.psychres.2016.08.044 27567192

[27] Kay SR Fiszbein A Opler LA. The positive and negative syndrome scale (PANSS) for schizophrenia. Schizophr Bull. (1987) 13:261–76. doi: 10.1093/schbul/13.2.261 3616518

[28] Cicero DC Kerns JG McCarthy DM. The aberrant salience inventory: A new measure of psychosis proneness. Psychol Assess. (2010) 22:688–701. doi: 10.1037/a0019913 20822281

[29] American Psychiatric Association. Diagnostic and statistical manual of mental disorders. 4th ed. Washington, DC: American Psychiatric Association (2000).

[30] Morosini PL Magliano L Brambilla L Ugolini S Pioli R. Development, reliability and acceptability of a new version of the DSM-IV Social and Occupational Functioning Assessment Scale (SOFAS) to assess routine social functioning. Acta Psychiatr Scand. (2000) 101:323–9. doi: 10.1034/j.1600-0447.2000.101004323.x 10782554

[31] Berzon RA Donnelly MA Simpson RL Simeon GP Tilson HH. Quality of life bibliography and indexes: 1994 update. Qual Life Res. (1995) 4:547–69. doi: 10.1007/BF00634750 8556015

[32] McGorry PD Hickie IB Yung AR Pantelis C Jackson HJ. Clinical staging of psychiatric disorders: a heuristic framework for choosing earlier, safer and more effective interventions. Aust N Z J Psychiatry. (2006) 40:616–22. doi: 10.1080/j.1440-1614.2006.01860.x 16866756

[33] Fusar-Poli P McGorry PD Kane JM. Improving outcomes of first-episode psychosis: an overview. World Psychiatry. (2017) 16:251–65. doi: 10.1002/wps.20446 PMC5608829 28941089

[34] Scott J Leboyer M Hickie I Berk M Kapczinski F Frank E. Clinical staging in psychiatry: A cross-cutting model of diagnosis with heuristic and practical value. Br J Psychiatry. (2013) 202(4):243–5. doi: 10.1192/bjp.bp.112.110858 23549937

[35] Maj M Os J De Hert M Gaebel W Galderisi S Green MF. The clinical characterization of the patient with primary psychosis aimed at personalization of management. World Psychiatry. (2021) 20:4–33. doi: 10.1002/wps.20809 33432763 PMC7801854

[36] Peralta V de Jalón EG Moreno-Izco L Peralta D Janda L Sánchez-Torres AM. A clinical staging model of psychotic disorders based on a long-term follow-up of first-admission psychosis: A validation study. Psychiatry Res. (2023) 322:115109. doi: 10.1016/j.psychres.2023.115109 36841052

[37] Fusar-Poli P Rutigliano G Stahl D Davies C De Micheli A Ramella-Cravaro V. Long-term validity of the At Risk Mental State (ARMS) for predicting psychotic and non-psychotic mental disorders. Eur Psychiatry. (2017) 42:49–54. doi: 10.1016/j.eurpsy.2016.11.010 28212505

[38] Fusar-Poli P Cappucciati M Bonoldi I Hui LMC Rutigliano G Stahl DR. Prognosis of brief psychotic episodes. JAMA Psychiatry. (2016) 73:211. doi: 10.1001/jamapsychiatry.2015.2313 26764163

[39] Leonhard K Beckmann H. Classification of Endogenous Psychoses and Their Differentiated Etiology. New York, NY: Springer (1999) p. 61–75.

[40] Marengo J. Classifying the courses of schizophrenia. Schizophr Bull. (1994) 20:519–36. doi: 10.1093/schbul/20.3.519 7973468

[41] World Health Organization. ICD-11: International classification of diseases (11th revision). (2022).

[42] American Psychiatric Association. Diagnostic and Statistical Manual of Mental Disorders. Fifth edition. Text Revision (DSM-5-TR (2022).

[43] Pallanti S. ICD and DSM: neuroplasticity and staging are still missing. CNS Spectr. (2016) 21:276–8. doi: 10.1017/S1092852916000146 27503571

[44] Stone WS Phillips MR Yang LH Kegeles LS Susser ES Lieberman JA. Neurodegenerative model of schizophrenia: Growing evidence to support a revisit. Schizophr Res. (2022) 243:154–62. doi: 10.1016/j.schres.2022.03.004 PMC9189010 35344853

[45] Ballester PL Romano MT de Azevedo Cardoso T Hassel S Strother SC Kennedy SH. Brain age in mood and psychotic disorders: a systematic review and meta-analysis. Acta Psychiatr Scand. (2022) 145:42–55. doi: 10.1111/acps.13371 34510423

[46] Seeman MV. Subjective overview of accelerated aging in schizophrenia. Int J Environ Res Public Health. (2022) 20:737. doi: 10.3390/ijerph20010737 36613059 PMC9819113

[47] Morin L Franck N. Rehabilitation interventions to promote recovery from schizophrenia: A systematic review. Front Psychiatry. (2017) 8:100. doi: 10.3389/fpsyt.2017.00100 28659832 PMC5467004

[48] Emsley R Chiliza B Asmal L. The evidence for illness progression after relapse in schizophrenia. Schizophr Res. (2013) 148:117–21. doi: 10.1016/j.schres.2013.05.016 23756298

[49] Lin D Joshi K Keenan A Shepherd J Bailey H Berry M. Associations between relapses and psychosocial outcomes in patients with schizophrenia in real-world settings in the United States. Front Psychiatry. (2021) 12:695672. doi: 10.3389/fpsyt.2021.695672 34764891 PMC8576536

[50] Kapadia M Desai M Parikh R. Fractures in the framework: limitations of classification systems inpsychiatry. Dialogues Clin Neurosci. (2020) 22:17–26. doi: 10.31887/DCNS.2020.22.1/rparikh 32699502 PMC7365290

[51] Dollfus S Brazo P. Clinical heterogeneity of schizophrenia. Psychopathology. (1997) 30:275–81. doi: 10.1159/000285059 9353856

[52] Jardri R Duverne S Litvinova AS Denève S. Experimental evidence for circular inference in schizophrenia. Nat Commun. (2017) 8:14218. doi: 10.1038/ncomms14218 28139642 PMC5290312

[53] Scott J Iorfino F Capon W Crouse J Nelson B Chanen AM. Staging 2.0: refining transdiagnostic clinical staging frameworks to enhance reliability and utility for youth mental health. Lancet Psychiatry. (2024) 11:461–71. doi: 10.1016/S2215-0366(24)00060-9 38643773

[54] Parker GB. Clinical staging of clinicians. Med J Aust. (2022) 217:587–8. doi: 10.5694/mja2.51780 PMC10098826 36385384

[55] Capon W Hickie IB Varidel M Prodan A Crouse JJ Carpenter JS. Clinical staging and the differential risks for clinical and functional outcomes in young people presenting for youth mental health care. BMC Med. (2022) 20:479. doi: 10.1186/s12916-022-02666-w 36514113 PMC9749194

[56] Scott J Henry C. Clinical staging models: From general medicine to mental disorders. BJPsych Adv. (2017) 23:292–9. doi: 10.1192/apt.bp.116.016436

[57] Berendsen S Van HL van der Paardt JW de Peuter OR van Bruggen M Nusselder H. Exploration of symptom dimensions and duration of untreated psychosis within a staging model of schizophrenia spectrum disorders. Early Interv Psychiatry. (2021) 15:669–75. doi: 10.1111/eip.13006 PMC8246761 32558322

[58] Singh SP. Outcome measures in early psychosis. Br J Psychiatry. (2007) 191:s58–63. doi: 10.1192/bjp.191.50.s58 18019046

[59] de Filippis R Gaetano R Schoretsanitis G Verde G Oliveti CA Kane JM. Clozapine management in schizophrenia inpatients: A 5-year prospective observational study of its safety and tolerability profile. Neuropsychiatr Dis Treat. (2021) 17:2141–50. doi: 10.2147/NDT.S312095 PMC8257059 34234440

[60] Aloi M de Filippis R Grosso Lavalle F Chiappetta E Viganò C Segura-Garcia C. Effectiveness of integrated psychological therapy on clinical, neuropsychological, emotional and functional outcome in schizophrenia: a RCT study. J Ment Heal. (2018) 29(5):524–31. doi: 10.1080/09638237.2018.1521948 30346226

[61] Kane JM Rubio JM. The place of long-acting injectable antipsychotics in the treatment of schizophrenia. Ther Adv Psychopharmacol. (2023) 13:204512532311572. doi: 10.1177/20451253231157219 PMC9989392 36895432

[62] Ostuzzi G Vita G Bertolini F Tedeschi F De Luca B Gastaldon C. Continuing, reducing, switching, or stopping antipsychotics in individuals with schizophrenia-spectrum disorders who are clinically stable: a systematic review and network meta-analysis. Lancet Psychiatry. (2022) 9:614–24. doi: 10.1016/S2215-0366(22)00158-4 35753323

[63] de Filippis R De Fazio P Gaetano R Steardo L Cedro C Bruno A. Current and emerging long-acting antipsychotics for the treatment of schizophrenia. Expert Opin Drug Saf. (2021) 20:771–90. doi: 10.1080/14740338.2021.1910674 33775184

[64] de Filippis R Staltari FA Aloi M Carbone EA Rania M Destefano L. Effectiveness of SGA-LAIs on clinical, cognitive, and social domains in schizophrenia: results from a prospective naturalistic study. Brain Sci. (2023) 13:577. doi: 10.3390/brainsci13040577 37190542 PMC10136480

[65] Addington D Anderson E Kelly M Lesage A Summerville C. Canadian practice guidelines for comprehensive community treatment for schizophrenia and schizophrenia spectrum disorders. Can J Psychiatry. (2017) 62:662–72. doi: 10.1177/0706743717719900 PMC5593248 28886669

[66] Norman R Lecomte T Addington D Anderson E. Canadian treatment guidelines on psychosocial treatment of schizophrenia in adults. Can J Psychiatry. (2017) 62:617–23. doi: 10.1177/0706743717719894 PMC5593243 28703017

[67] Grover S Chakrabarti S Kulhara P Avasthi A. Clinical practice guidelines for management of schizophrenia. Indian J Psychiatry. (2017) 59:19. doi: 10.4103/0019-5545.196972 PMC5310098 28216783

