# Supplementary material for: Applying a clinical staging model in patients affected by schizophrenia spectrum disorder
Source: Front Psychiatry. 2024 Jul 16;15:1387913. doi: 10.3389/fpsyt.2024.1387913 (PMC11287066; doi:10.3389/fpsyt.2024.1387913)
Supplement: Supplementary Table 1 — Clinical staging guidelines. [file Table_1.pdf]

Supplementary Table 1. Clinical Staging guidelines.

Stage	Definition
1	**Mild/moderate psychotic symptoms**<br>The individual experiences pre-psychotic or mild/moderate psychotic symptoms that do not reach the full significance of a full-blown episode and do not irreversibly impact personal functioning.
2A	**Episodic course with full remission (single episode)**<br>After a first episode of psychosis, the individual experiences a complete remission of symptoms and returns to the premorbid level of functioning. There are no subsequent psychotic or major mood episodes up to the follow-up visit.
2B	**Episodic course with partial remission (multiple episodes)**<br>After experiencing more than one episode of psychosis, the individual achieves a partial remission of symptoms. Although the individual does not return to the full premorbid level of functioning, there is no significant impairment.
3A	**Episodic course with partial and stable remission**<br>The individual may exhibit residual symptoms and/or a decline in premorbid functioning in one or more areas between episodes. These residual symptoms and functional declines, with remission compared to the acute episodes, tend to remain stable over the course of the illness.
3B	**Episodic course with partial remission and progressive course**<br>During alternating episodes, symptom level tend to increase, and/or functioning tends to decrease in one or more areas. Even if this progression does not follow a continuous trend, a general pattern of worsening symptoms and declining functioning is evident over the course of the illness.
4	**Chronic/continuous course**<br>Throughout the illness, there is a continuous and stable pattern of symptoms and functional decline that treatment does not significantly influence. Although there may be periods of improvement/worsening/stable pattern of symptoms and/or functioning, the severe impairment remains predominant over the course of the illness.

Only the current illness state at the time of clinical assessment and evaluation should be considered when rating the stage.

Staging levels serve as ideal representations of the current course and outcomes of psychotic disorders. It is relatively common for different course patterns to occur successively over the long term and change quickly after treatment. In such cases, the pattern with the longest duration throughout the illness takes precedence, especially if it has been present during the most recent years of follow-up.

An episode or relapse is defined as a clear aggravation in symptoms or functioning those results in a change in the treatment regimen and possibly admission to a more specialized service and/or needs hospitalization.
