# Supplementary material for: Applying a clinical staging model in patients affected by schizophrenia spectrum disorder
Source: Front Psychiatry. 2024 Jul 16;15:1387913. doi: 10.3389/fpsyt.2024.1387913 (PMC11287066; doi:10.3389/fpsyt.2024.1387913)
Supplement: Supplementary Table 2 — Mean differences between stages according to psychopathological variables. [file Table_2.pdf]

Supplementary Table 2. Mean differences between stages according to psychopathological variables.

	**S1**		**S2A**		**S2B**		**S3A**		**S3B**		**S4**		**ANOVA**	
	Mean	SD	Mean	SD	Mean	SD	Mean	SD	Mean	SD	Mean	SD	F	*p*
**PANSS Positive**	9.0	5.4	13.6	8.8	17.5	6.4	18.4	11.0	14.8	6.8	19.3	8.9	1.17	.33
**PANSS Negative**	9.0	4.3	17.4	9.7	21.7	7.3	21.8	9.7	20.0	9.9	21.7	9.7	.61	.69
**PANSS General**	27.0	11.4	35.4	13.8	40.8	8.6	44.6	13.3	38.6	9.3	45.5	10.2	2.23	.06
**PANSS Total**	45.0	33.8	66.4	32.0	81.4	16.3	86.2	30.5	74.9	20.6	88.7	21.2	1.74	.43
**GAF**	85.0	22.4	58.8	18.2	57.4	13.3	48.9	11.9	47.8	9.2	44.9	12.6	4.68	**<.001**
**CTQ**	61.0	7.2	48.7	15.9	53.8	12.0	55.1	10.6	55.2	9.5	58.3	6.7	1.64	.16
**ASI**	12.0	7.2	17.1	4.0	17.9	6.9	19.3	7.5	18.3	6.9	19.7	4.8	1.12	.35
**QoL Scale**	91.3	1.5	72.4	27.1	50.3	22.9	39.6	6.5	39.5	17.0	34.3	12.3	12.95	**<.001**
**PSP Scale**	75.0	21.5	58.6	19.9	54.1	10.9	49.4	13.3	46.1	10.8	43.5	13.8	3.22	.01

ASI: Aberrant Salience Inventory; CTQ: Childhood Trauma Questionnaire; GAF: Global Assessment of Functioning; PANSS: Positive and Negative Symptom Scale; PSP: Personal and Social Performance; QoL: Quality of Life; S: stage; SD: Standard deviation.
