# Supplementary material for: Applying a clinical staging model in patients affected by schizophrenia spectrum disorder
Source: Front Psychiatry. 2024 Jul 16;15:1387913. doi: 10.3389/fpsyt.2024.1387913 (PMC11287066; doi:10.3389/fpsyt.2024.1387913)
Supplement: Supplementary Material 3 — Methodology to assign the clinical stage. [file DataSheet_1.pdf]

Supplementary Material 3. Methodology to assign the clinical stage.

General methodology

STEP 1: Evaluation of non-modifiable variables

- Sociodemographic variables

The first step to carry out an adequate clinical staging of schizophrenia and collect as much anamnestic information as possible on the patient and his clinical history. These include but are not limited to: gender, age, personal and family psychiatric history with particular relevance to psychotic diseases, parental socioeconomic status, childhood trauma and adversities, level of premorbid functioning, educational qualifications, marital status, urbanicity level, age at onset, duration of illness, number of suicide attempts, number of voluntary and compulsory hospitalizations, smoking and alcohol habits, illicit drugs use and abuse, previous psychiatric treatments, previous psychiatric diagnoses, number of psychotic episodes.

This step can be conducted with a free interview, through collection of information from the medical record or chart, or through validated tests.

Among the most used structured assessment the Comprehensive Assessment of Symptoms and History (CASH) represents one of the feasible options. A major advantage of the CASH is its broad descriptive coverage of lifetime history through the Life Chart Interview (LCI), including symptoms and diagnoses. Additionally, CASH includes the Scale for the Assessment of Positive Symptoms (SAPS) and the Scale for the Assessment of Negative Symptoms (SANS). While, for some relevant variables not included in the CASH, other specific assessment instruments may be employed to integrate the interview.

All variables collected in this, and next step can be assessed using multiple information sources, including interviews with participants, clinical records, first-degree relatives, significant others, and, if necessary, information from the primary physician.

STEP 2: Evaluation of modifiable variables

- Clinical and functioning evaluation

Psychopathology severity during the current illness state was traced using the ASI, CTQ-SF, GAF, PANSS, PSP, QoLS for aberrant salience, childhood trauma and adversities, psychotic symptoms, disorganization, negative symptoms, global functioning and quality of life during the current evaluation. Severity and frequency scores were combined to define each dimension gravity.

STEP 3: Current clinical assessment

-Summarizing relevant data collected

A summary of the data obtained is carried out, marking the most relevant and severe ones by means of the clinical assessment and including lifetime ratings for psychopathology, functioning, illness-extension variables, mental health service utilization and dose-years of psychiatric medication.

Moreover, we evaluated the illness-extension variables included the DSM-5 diagnosis, number of psychotic or major mood episodes, number of suicide attempts, and severity of drug abuse. Still, mental health service utilization included the number of psychiatric emergency visits, number of admissions to diverse psychiatric hospital wards and participation in a formal program of psychosocial rehabilitation or psychotherapy treatments. Lifetime medication use for antipsychotics, anticholinergics, antidepressants, mood stabilizers and benzodiazepines, considering both years on specific medications and dosages.

STEP 4: Assignment of a clinical stage

-Stages: 1-2A-2B-3A-3B-4

Based on the information collected and the current assessment of the patient, the guidelines presented in Supplementary Table 1 are followed to assign the patient to a precise stage that best describes the current psychopathological condition.
